# Virulence-associated genes analysis of carbapenemase-producing *Escherichia coli* isolates

**DOI:** 10.1371/journal.pone.0266787

**Published:** 2022-05-10

**Authors:** Nabi Jomehzadeh, Fateme Jahangirimehr, Sina Ahmadi Chegeni

**Affiliations:** 1 Department of Microbiology, School of Medicine, Abadan University of Medical Sciences, Abadan, Iran; 2 Department of Biostatistics and Epidemiology, Ahvaz Jundishapur University of Medical Sciences, Ahvaz, Iran; Suez Canal University, EGYPT

## Abstract

Carbapenem-resistant *Escherichia coli* has emerged as a major public health issue across the world. This study was aimed to determine the virulence content and phylogenetic groups of carbapenemase-producing *E*. *coli* isolates in southwest Iran. One hundred and fifty-two non-duplicate *E*. *coli* isolates were collected from various clinical samples. Antibiotic susceptibility and minimum inhibitory concentrations (MIC) were determined according to the Clinical and Laboratory Standards Institute (CLSI) guidelines by Kirby-Bauer disc diffusion and agar dilution methods. Phenotypic screening of carbapenemase enzymes was performed by modified Hodge test (MHT). Detection of carbapenemase genes, phylogenetic groups, and virulence-associated genes were also performed by the PCR assay. The highest and lowest resistance rates were observed against mezlocillin (70.4%) and doripenem (13.1%), respectively. Out of 28 isolates that were resistant to carbapenem antibiotics, 12 (7.9%) strains were phenotypically carbapenemase producers. The *bla*_OXA-48_ was the predominant carbapenemase gene, detected in 58.3% of isolates, followed by *bla*_IMP_ (41.7%) and *bla*_NDM_ (8.3%). None of the isolates harbored *bla*_VIM_ and *bla*_KPC_ genes. Among the twelve carbapenemase-producing strains, urinary isolates were mostly classified into B2 (41.7%) and D (25%) phylogenetic groups, while other clinical isolates belonged to B1 (25%) and A (8.3%) groups. The frequency of virulence-associated genes was also investigated in all isolates and ranged from 6.6% for *hly* to 75% for *fimA*. The emergence of carbapenemase-producing strains is a growing concern to public health. Therefore, the proper implementation of monitoring programs is crucial for limiting their dissemination.

## Introduction

Multidrug resistance has been increased all over the world that is considered a public health threat [1]. Several recent investigations reported the emergence of multidrug-resistant (MDR) bacterial pathogens from different origins including humans, birds, cattle, and fish that increase the need for routine application of the antimicrobial susceptibility testing to detect the antibiotic of choice as well as the screening of the emerging MDR strains [2–9]. Most members of the Enterobacteriaceae and particularly *Escherichia coli* strains are of special importance due to their high prevalence in the community, ability to cause various infections, and high resistance to most antibiotics [10]. *E*. *coli* is an opportunistic pathogen in the gut of healthy individuals. Some strains of this bacterium can colonize other tissues and host organs and become extraintestinal pathogenic *E*. *coli* (ExPEC) [11]. ExPEC using an arsenal of virulence-associated factors can overcome host defense systems and cause serious diseases such as sepsis, meningitis, pneumonia, urinary tract infections, cystitis, and pyelonephritis [12]. The pathogenicity of ExPEC depends on the various virulence factors, including; adhesins, toxins, iron-acquisition factors, and invasins which are encoded by the several virulence genes: *fimA* (type 1 fimbriae), *papGIII*, *papC* (P fimbriae), *sfa* (S fimbriae), *afa* (afimbrial adhesins), *cnf1* (cytotoxic necrotizing factor 1), *sat* (secreted autotransporter toxin), *hly* (hemolysins), *aer* (aerobactin), *iutA* (aerobactin siderophore receptor), and *iroN* (salmochelin siderophore receptor) [12, 13]. Generally, *E*. *coli* strains are categorized into four major phylogenetic groups (A, B1, B2, and D). According to epidemiological studies, ExPEC strains are often sorted as phylogroup D or B2, whereas commensal strains are frequently classified as phylogroup A or B1. However, horizontal genetic transmission processes allow the exchange of virulence-associated genes among phylogroups, which may confer the emergence of highly pathogenic strains belonging to phylogroups A or B1 [14].

Due to growing resistance to broad-spectrum antibiotics including fluoroquinolones, third-generation cephalosporins, and aminoglycosides, the carbapenems have progressively become the last line for treatment of life-threatening infections [15]. However, with the dramatic increase in carbapenems usage, the emergence of carbapenem-resistant species has become a mounting public health problem around the world [16]. There are various mechanisms for resistance to carbapenems, one of the main of which is the production of carbapenemase enzymes. The most common carbapenemases identified in Enterobacteriaceae are class A (KPC), class B (NDM, IMP, and VIM), and class D (type OXA-48). The widespread occurrence of carbapenemase-producing *E*. *coli* could trigger a new epidemiological crisis, similar to that caused by extended-spectrum β-lactamases [17]. Although several studies have been performed on the prevalence of carbapenem-resistant *E*. *coli* in different parts of Iran, our data in the southwestern region are very limited. Thus, this study was aimed to investigate the frequency of carbapenemase-producing *E*. *coli* isolates and their correlation with phylogenetic background and Virulence-associated genes.

## Materials and methods

### Ethics approval

The study protocol has been under the Helsinki Declaration and received ethical approval from the Institutional Ethics Committee of Abadan University of Medical Sciences (Ethical Code: IR.ABADANUMS.REC.1399.169).

### Bacterial isolation and identification

From Jan 2021 to Sep 2021, 152 nonduplicate *E*.*coli* isolates were collected from clinical specimens (including urine, sputum, wound, and blood) of patients admitted to affiliated hospitals of the Abadan University of Medical Sciences. All isolates were accurately identified by performing standard methods [18]. Briefly, the specimens were inoculated in MacConkey broth (Merck, Darmstadt, Germany) and incubated at 37°C for 24 h. A loopful of broth culture was subsequently cultured on Eosin Methylene Blue (EMB; Biolife Italiana, Italy) and MacConkey agar. All grown lactose-fermenting colonies were identified via bacteriological tests (such as hemolytic activity on blood agar, motility test, and Gram staining result) and conventional biochemical tests including triple sugar iron agar, oxidase, catalase, production of lysine decarboxylase, citrate utilization test, Sulfur Indole Motility (SIM), Methyl Red & Vogues-Proskauer (MR-VP), and urease test [18, 19]. Finally, purified isolates stored in trypticase soy broth (Merck, Darmstadt, Germany) containing 20% glycerol at -70°C until further use.

### Antimicrobial susceptibility testing

The antibiotic susceptibility testing was performed for *E*.*coli* isolates by the Kirby-Bauer disc diffusion method under the Clinical and Laboratory Standards Institute (CLSI) guidelines [20] for the following standard antibiotics (Roscoe, Taastrup, Denmark): Cefepime (FEP; 30 μg) Imipenem (IMP; 10 μg), Ampicillin/Sulbactam (SAM; 20 μg), Ertapenem (ETP; 10 μg), Meropenem (MEM; 10 μg), Aztreonam (ATM; 30 μg), Doripenem (DOR; 10 μg), and Mezlocillin (MEZ; 30 μg). *E*. *coli* ATCC 25922 was used as a quality stander strain. In addition, for carbapenem non-susceptible isolates, resistance to imipenem was evaluated by minimum inhibitory concentration (MIC) with a standard agar dilution test [20]. All studied isolates were also classified into MDR (non-susceptibility to at least one agent in ≥ three antimicrobial families), extensively drug-resistant (XDR; non-susceptibility to ≥ one agent in all but ≤ two antimicrobial families) and pandrug-resistant (PDR; non-susceptibility to all antimicrobial classes) as previously described [21].

### Phenotypic detection of carbapenemase

For confirmation of carbapenemase production, all imipenem-resistant strains were screened by the Modified Hodge test (MHT) according to the CLSI guidelines [20]. In this method, an overnight suspension of *E*. *coli* ATCC 25922 adjusted to the turbidity of the 0.5 McFarland was prepared and cultured uniformly on Müller-Hinton agar (MHA; Merck Co., Darmstadt, Germany) containing 70 μg per ml of ZnSO4. A carbapenem disk was placed in the center of the plate, and the microorganism suspected of producing carbapenemase was drawn in a straight line from the edge of the disk to the sides of the plate. The clover leaf-shaped inhibition zone formation around the central disc was considered as carbapenemase production.

### Detection of carbapenemase genes by Multiplex PCR

Bacterial DNA was extracted using the simple boiling method [22]. The presence of carbapenemase genes including (*bla*_NDM_, *bla*_VIM_, *bla*_KPC_, *bla*_IMP_, and *bla*_OXA-48_) was determined using Multiplex PCR assay as previously described [23]. The PCR products were run on 1.8% agarose gel stained with 0.5 μg mL-1 ethidium bromide.

### Molecular characterization of phylogenetic groups

All isolates were phylogenetically categorized into four main groups (A, B1, B2, and D) using triplex PCR assay as described by Clermont et al. [24]. To confirm the presence of the amplified fragment, PCR product electrophoresis on 2% agarose gel along with molecular size marker 100 bp (Ampliqon, Denmark) was examined.

### Multiplex PCR for virulence genes

In this study, 11 different virulence gene factors (*fimA*, *papC*, *papGIII*, *aer*, *sat*, *afa*, *sfa*, *cnf-1*, *hly*, *iutA*, and *iroN*) were assessed by PCR method using a thermal cycler (Bio-Rad Laboratories, Inc.). The multiplex PCR reaction was performed in 25 μL volumes containing 3 μl of DNA template, 1 μl of each specific primer, 12.5 μL of Master Mix Red (Ampliqon, Denmark), and 7.5 μl of double-distilled water. The sequences of used primers and amplification conditions are presented in Table 1 [25–27]. The PCR products were analyzed by 1.8% agarose gel electrophoresis in 1x TBE buffer (0.89 M Tris-Borate, 0.02 M EDTA, pH 8.3), stained with ethidium bromide (SinaClon BioScience Co., Iran), and visualized by using UV light.

**Table 1:** List of primers sequences used in this study.

Gene	Primer Sequence (5´→3´)	Size of Product	Amplification	Reference
*bla*_NDM_	GGTTTGGCGATCTGGTTTTC CGGAATGGCTCATCACGATC	621	94°C, 10 min; 36 cycles of 94°C for 30 s, 52°C, 40 s, 72°C, 50 s, final extension 72°C, 5 min.	23
*bla*_VIM_	GATGGTGTTTGGTCGCATA CGAATGCGCAGCACCAG	390		
*bla*_KPC_	CGTCTAGTTCTGCTGTCTTG CTTGTCATCCTTGTTAGGCG	798		
*bla*_IMP_	GGAATAGAGTGGCTTAAYTCTC GGTTTAAYAAAACAACCACC	232		
*bla*_OXA-48_	GCGTGGTTAAGGATGAACAC CATCAAGTTCAACCCAACCG	438		
*fimA*	GTTGTTCTGTCGGCTCTGTC ATGGTGTTGGTTCCGTTATTC	447	94°C, 3 min; 30 cycles of 94°C for 1 min, 55°C, 30 s, 72°C, 1 min, final extension 72°C, 8 min.	25
*papGIII*	CATTTATCGTCCTCAACTTAG AAGAAGGGATTTTGTAGCGTC	482		
*sat*	ACTGGCGGACTCATGCTGT AACCCTGTAAGAAGACTGAGC	387		
*afa*	GCTGGGCAGCAAACTGATAACTCTC CATCAAGCTGTTTGTTCGTCCGCCG	750	94°C, 3 min; 30 cycles of 94°C for 1 min, 63°C, 30 s, 72°C, 1 min, final extension 72°C, 8 min.	26
*sfa*	CTCCGGAGAACTGGGTGCATCTTAC CGGAGGAGTAATTACAAACCTGGCA	410		
*hly*	AACAAGGATAAGCACTGTTCTGGCT ACCATATAAGCGGTCATTCCCGTCA	1177		
*cnf-1*	AAGATGGAGTTTCCTATGCAGGAG CATTCAGAGTCCTGCCCTCATTATT	498		
*aer*	TACCGGATTGTCATATGCAGACCGT AATATCTTCCTCCAGTCCGGAGAAG	602		
*papC*	GACGGCTGTACTGCAGGGTGTGGCG ATATCCTTTCTGCAGGGATGCAATA	328		
*iutA*	GGCTGGACATCATGGGAACTGG CGTCGGGAACGGGTAGAATCG	300	94°C, 3 min; 30 cycles of 94°C for 1 min, 58°C, 30 s, 72°C, 1 min, final extension 72°C, 8 min.	27
*iroN*	AAGTCAAAGCAGGGGTTGCCCG GACGCCGACATTAAGACGCAG	665		

### Statistical analysis

The statistical analysis was performed using the Statistical Package for Social Sciences Statistics software (SPSS; IBM, Chicago, IL, USA) version 21.0. For the objectives of this study, Fisher’s exact test or Chi-square test were used for comparison and *P*-value < 0.05 was considered statistically significant. To examine the relationship between the two nominal variables, a Phi correlation test was used.

## Results

### Phenotypic characteristics of the recovered isolates

Of the 152 *E*. *coli* strains, 129 (84.9%) were isolated from urine, 8 (5.3%) from the wound, 9 (5.9%) from blood, and 6 (3.9%) from sputum. All collected isolates have been identified as *E*. *coli* according to their morphological and biochemical characteristics. The isolates appeared as motile gram-negative rods under a microscope, and after growing on MacConkey agar gave distinct pink colonies due to the fermentation of lactose. They also had hemolytic colonies on blood agar and typical shiny metallic colonies on EMB. Isolates were positive for biochemical tests of catalase, lactose fermentation, MR, and indole. However, their oxidase, lysine decarboxylase, VP, citrate, H2S production, and urease tests were negative.

### Antimicrobial susceptibility testing

**Fig 1:**
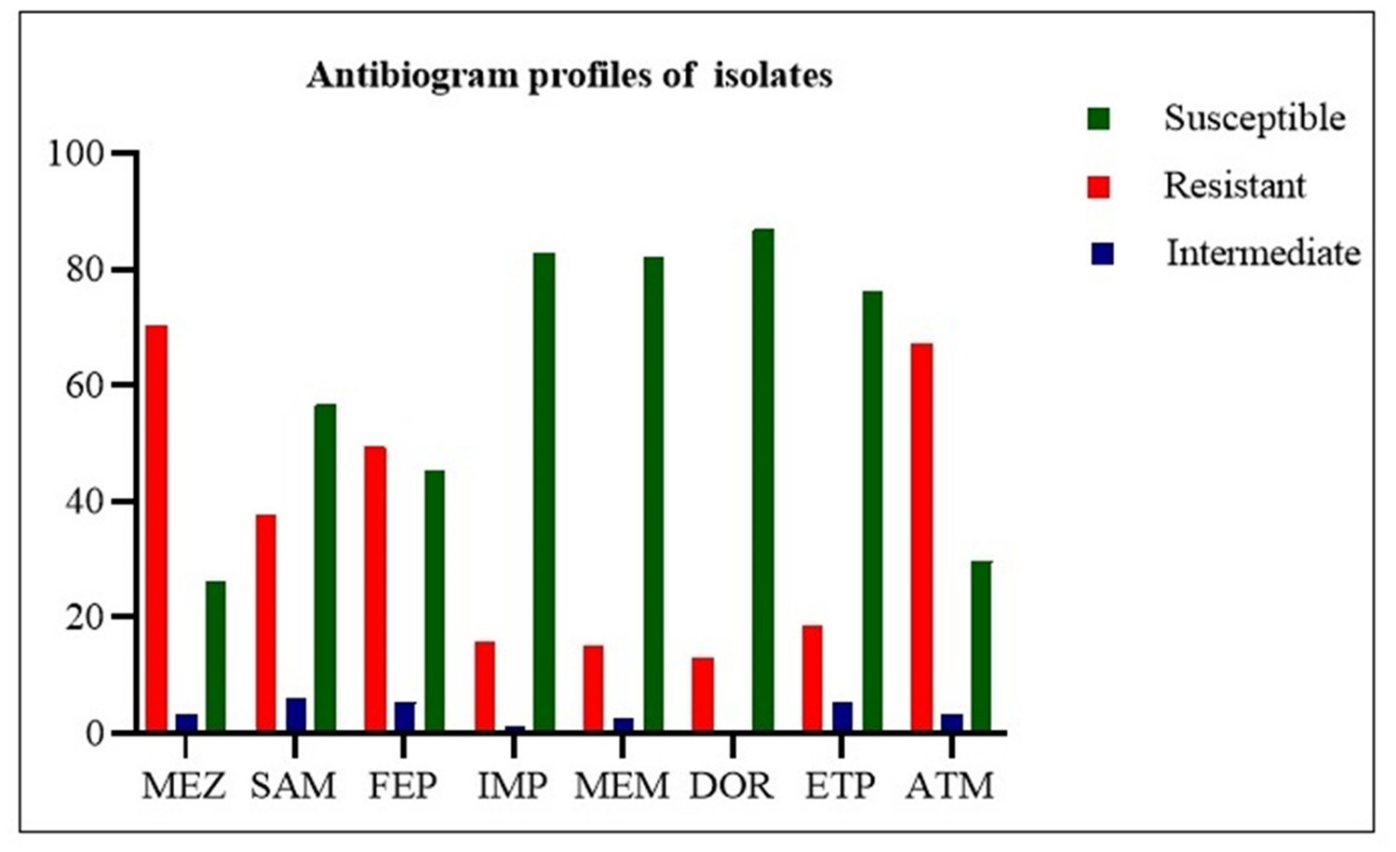
Antimicrobial resistance profile of the 152 *E*. *coli* isolates.

All studied isolates showed significant resistance to tested antibiotics. The highest resistance rates were observed in mezlocillin (70.4%, 107 isolates) and aztreonam (67.1%, 102 isolates). In contrast, 13.1% (20 isolates) and 15.1% (23 isolates) of the isolates were resistant to doripenem and meropenem, respectively (Table 2, Fig 1). The MIC was evaluated for 26 imipenem non-susceptible isolates and the results showed 12 were resistant to 4- >128 μg/ml of imipenem (Table 5). In total, 28 (18.4%) isolates were resistant to at least one of the carbapenem drugs. MDR phenomena were found in 104 (68.4%) isolates, among which, only 12 were harboring various carbapenemases genes (Table 3). Correlation analysis was performed between different phenotypic MDR patterns and the carbapenems resistance genes. The derived results disclosed strong positive correlations between *bla*_NDM_ gene and DOR (phi = 0.704); *bla*_OXA-48_ gene, MEM (phi = 0.781), and IMP (phi = 0.744); *bla*_IMP_ gene, MEM (phi = 0.654), and IMP (phi = 0.622) (Table 4).

**Table 2:** Antimicrobial resistance profile of the 152 *E*. *coli* isolates.

Antibiotic classes	Antimicrobials	Resistant (%)	Intermediate (%)	Susceptible (%)
Penicillins	Mezlocillin	107 (70.4)	5 (3.3)	40 (26.3)
	Ampicillin/Sulbactam	57 (37.5)	9 (5.9)	86 (56.6)
Cephalosporins	Cefepime	75 (49.3)	8 (5.3)	69 (45.4)
Carbapenems	Imipenem	24 (15.8)	2 (1.3)	126 (82.9)
	Meropenem	23 (15.1)	4 (2.6)	125 (82.2)
	Doripenem	20 (13.1)	0	132 (86.8)
	Ertapenem	28 (18.4)	8 (5.3)	116 (76.3)
Monobactams	Aztreonam	102 (67.1)	5 (3.3)	45 (29.6)

**Table 3:** Frequency of the antimicrobial resistance profile and the resistance genes among all *E*. *coli* isolates.

No. (%) of strains	Type of resistance	Phenotypic MDR	Resistance genes
83 (54.6)	MDR	Penicillins: mezlocillin, ampicillin/sulbactam Carbapenems: ertapenem Monobactams: aztreonam	ND
9 (5.9)	MDR	Penicillins: mezlocillin, ampicillin/sulbactam Cephalosporins: cefepime Monobactams: aztreonam	ND
29 (19.1)	Resistant	Penicillins: mezlocillin, ampicillin/sulbactam Carbapenems: ertapenem	ND
19 (12.5)	Resistant	Penicillins: mezlocillin Monobactams: aztreonam	ND
4 (2.6)	MDR	Carbapenems: ertapenem, imipenem, meropenem Monobactams: aztreonam Cephalosporins: cefepime	*bla*_IMP_
6 (3.9)	MDR	Penicillins: mezlocillin, ampicillin/sulbactam Carbapenems: ertapenem, imipenem, meropenem Cephalosporins: cefepime	*bla*_OXA-48_
1 (0.6)	MDR, Possible XDR	Penicillins: ampicillin/sulbactam Carbapenems: ertapenem, imipenem, doripenem Monobactams: aztreonam Cephalosporins: cefepime	*bla*_NDM_
1 (0.6)	MDR, Possible XDR	Penicillins: mezlocillin, ampicillin/sulbactam Carbapenems: imipenem, meropenem, ertapenem, doripenem Cephalosporins: cefepime Monobactams: aztreonam	*bla*_IMP_, *bla*_OXA-48_

ND, not detected.

**Table 4:** The correlation between various phenotypic MDR patterns and the carbapenems resistance genes.

	*bla*_NDM_	*bla*_OXA-48_	*bla*_IMP_	DOR	MEM	ETP	IMP	ATM	MEZ	SAM	FEP
*bla*_NDM_	1.00	-0.026	-0.022	**0.704**	-0.034	0.030	0.273	0.024	-0.439	0.020	0.196*
	-	0.788	0.822	<0.0001	0.731	0.758	0.005	0.805	<0.0001	0.842	0.044**
*bla*_OXA-48_		1.00	0.120	0.242	**0.781**	0.082	**0.744**	-0.921	0.060	0.053	0.535
		-	0.221	0.013	<0.0001	0.402	<0.0001	<0.0001	0.540	0.586	<0.0001
*bla*_IMP_			1.00	0.296	**0.654**	0.068	**0.622**	0.055	-0.790	-0.890	0.447
			-	0.002	<0.0001	0.483	<0.0001	0.573	<0.0001	<0.0001	<0.0001
DOR				1.00	0.180	0.043	0.388	0.034	-0.296	0.028	0.279
				-	0.065	0.662	<0.0001	0.725	0.002	0.776	0.004
MEM					1.00	0.105	0.952	-0.720	-0.508	-0.582	0.684
					-	0.283	<0.0001	<0.0001	<0.0001	<0.0001	<0.0001
ETP						1.00	0.110	-0.075	-0.068	-0.061	-0.612
						-	0.260	0.440	0.483	0.532	<0.0001
IMP							1.00	-0.685	-0.622	-0.554	0.718
							-	<0.0001	<0.0001	<0.0001	<0.0001
ATM								1.00	-0.55	-0.049	-0.492
								-	0.573	0.616	<0.0001
MEZ									1.00	0.890	-0.447
									-	<0.0001	<0.0001
SAM										1.00	-0.398
										-	<0.0001
FEP											1.00
											-

* Phi coefficient

** Approximate Significance

### Phenotypic detection of carbapenemase

After performing the phenotypic MHT, 12 (7.9%) isolates showed positive tests and were confirmed as carbapenemase producers.

### PCR- based detection of carbapenem-resistance genes

Based on PCR results, all twelve imipenem non-susceptible strains carried at least one carbapenemase-related gene (Table 5). The *bla*_OXA-48_ was the most prevalent gene, detected in 58.3% (7/12) of isolates, followed by *bla*_IMP_ (41.7%, 5/12) and *bla*_NDM_ (8.3%, 1/12). Also, the coharboring of two genes, *bla*_OXA-48_, and *bla*_IMP_ were observed in one isolate. None of the strains carried *bla*_VIM_ and *bla*_KPC_ genes.

**Table 5:** Carbapenem resistance pattern, phylogenetic grouping, and virulence genes profiles of 12 carbapenemase-producers isolates.

Strains (n = 12)	Sample Type	Carbapenemase Genes	Phylogenetic group	Virulence factors	CRP	IMP MIC (μg /ml)
E05	Urine	*bla*_IMP_	B2	*fimA*	IMP	16
E12	Urine	*bla*_OXA-48_	D	*papC*, *cnf-1*, *fimA*	IMP, ETP	32
E14	Sputum	*bla*_IMP_	D	*iroN*, *iutA*, *afa*	MER	8
E52	Wound	*bla*_NDM_	B1	*sat*, *afa*, *aer*, *fimA*, *papGIII*, *iroN*, *cnf-1*, *iutA*	IMP, ETP, DOM	>128
E37	Urine	*bla*_IMP_	B2	*fimA*, *papGIII*, *iutA*	ETP	4
E10	Urine	*bla*_OXA-48_	B2	*fimA*, *sfa*, *hly*	IMP	16
E28	Urine	*bla*_OXA-48_	B2	*cnf-1*, *fimA*, *sfa*, *hly*	ETP	16
E61	Wound	*bla*_OXA-48_	B1	*sat*, *afa*, *fimA*, *iroN*, *cnf-1*, *iutA*	IMP, MER, ETP	64
E73	Blood	*bla*_IMP_	A	*afa*, *aer*, *fimA*, *iutA*	MER	8
E77	Wound	*bla*_IMP_, *bla*_OXA-48_	B1	*sfa*, *hly*, *aer*, *fimA*, *papC*, *iroN*, *cnf-1*, *iutA*, *afa*	IMP, MER, ETP, DOM	>128
E65	Urine	*bla*_OXA-48_	B2	*fimA*, *sfa*, *papGIII*, *iutA*	IMP, MER	16
E23	Urine	*bla*_OXA-48_	D	*cnf-1*, *fimA*, *sfa*, *papGIII*, *iutA*	IMP	4

CRP: Carbapenem-resistant pattern, IMP: Imipenem, MER: Meropenem, ETP: Ertapenem, Doripenem: DOM, The resistance breakpoint (CLSI) for imipenem is MIC ≥4 mg/ml.

### PCR- based detection of virulence-determinant genes

**Fig 2:**
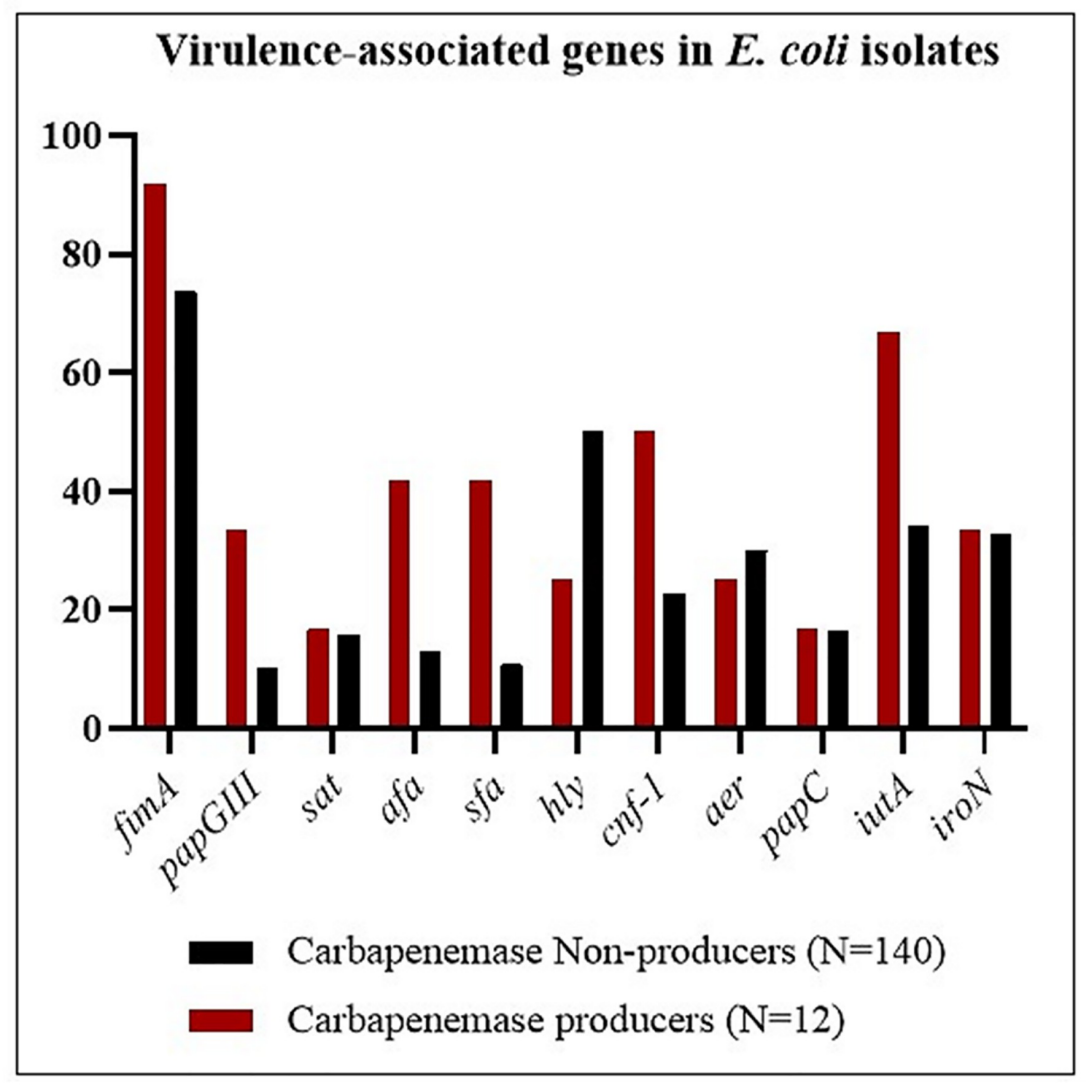
Distribution of virulence factors genes among the retrieved strains (n = 152).

**Fig 3:**
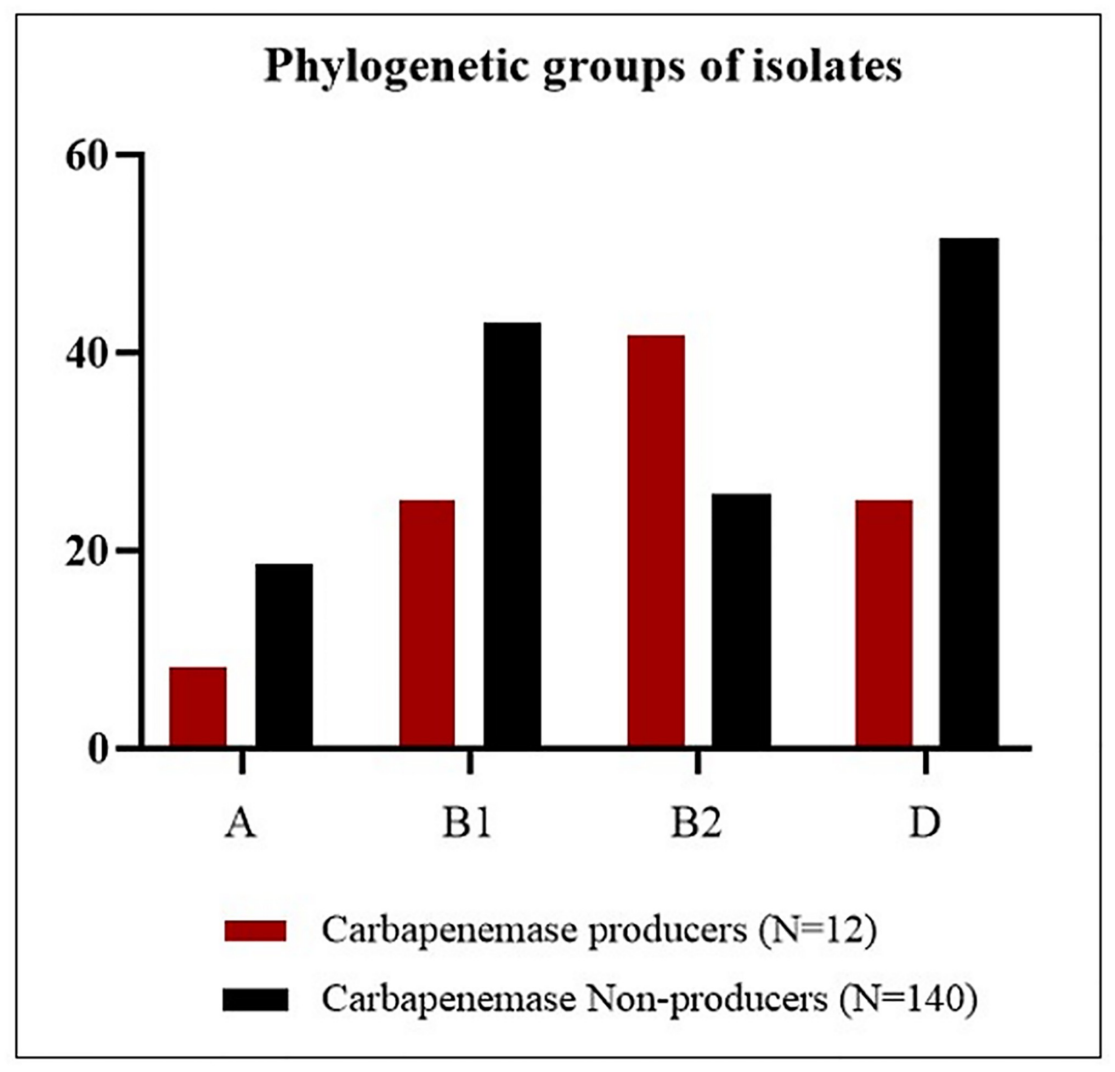
Distribution of phylogenetic groups among the retrieved strains (n = 152).

The frequency of the four major phylogroups (A, B1, B2, and D) in carbapenemase-producers isolates was differed based on the findings of the triplex PCR test. Among all 152 *E*. *coli* isolates, the predominant phylogenetic groups were D (49.3%) followed by B2 (27%) (Table 6, Fig 2). In the carbapenemase-producers, the urinary isolates have belonged to phylogroups B2 (41.7%) and D (25%). Also, other clinical isolates belonged to groups B1 (25%) and A (8.3%) (Table 5). The results showed significant differences in phylogroups B1 (p = 0.024) between carbapenemase producers and non-producers. In this study, the frequency of 11 virulence factors for *E*.*coli* isolates was investigated and ranged from 6.6% (*hly*) to 75% (*fimA*). Based on data from the multiplex PCR results, 91.7% (11/12) of imipenem non-susceptible strains were positive for *fimA*. The correlation between the frequency of virulence genes and carbapenemase production was not statistically significant except for *papGIII*, *afa*, *sfa*, *hly*, *cnf-1*, and *iutA*. The distribution and correlation of the virulence-associated genes are shown in detail in Table 6 and Fig 3. Remarkably, the strains isolated from the wound specimen were not only resistant to most carbapenems tested and had the highest MIC, but also contained several virulence factors genes.

**Table 6:** Distribution of virulence genes and phylogenetic groups among carbapenemase producer and non-producer isolates.

Virulence genes	Carbapenemase		Total (N = 152) (%)	*p-value*
	Producers (N = 12) (%)	Non-producers (N = 140) (%)		
*fimA*	11 (91.7)	103 (73.6)	114 (75)	0.296
*papGIII*	4 (33.3)	14 (10)	18 (11.8)	**0.038**
*sat*	2 (16.7)	22 (15.7)	24 (15.8)	>0.999
*afa*	5 (41.7)	18 (12.8)	23 (15.1)	**0.020**
*sfa*	5 (41.7)	15 (10.7)	20 (13.1)	**0.010**
*hly*	3 (25)	7 (5)	10 (6.6)	**0.033**
*cnf-1*	6 (50)	32 (22.8)	38 (25)	**0.074**
*aer*	3 (25)	42 (30)	45 (31.7)	>0.999
*papC*	2 (16.7)	23 (16.4)	25 (16.4)	>0.999
*iutA*	8 (66.7)	48 (34.3)	56 (36.8)	**0.049**
*iroN*	4 (33.3)	46 (32.9)	50 (32.9)	>0.999
**Phylogenetic group**				
A	1 (8.3)	25 (17.8)	26 (17.1)	0.692
B1	3 (25)	6 (4.3)	9 (5.9)	**0.024**
B2	5 (41.7)	36 (25.7)	41 (27)	0.314
D	3 (25)	72 (51.5)	75 (49.3)	0.130

Numbers in bold are statistically significant.

## Discussion

Carbapenems are commonly used in clinical settings to treat MDR gram-negative bacterial infections owing to their broad spectrum of antibacterial activity [15]. Yet, several monitoring programs claim that the overuse of these antibiotics and the emergence of carbapenem-resistant organisms have become a major global health concern [28]. Eastern Mediterranean countries, including Iran, have the highest risk of antimicrobial resistance, and carbapenem-resistant *E*. *coli* strains are on the WHO list of global priority pathogens, which is classified as critical [29]. To best our knowledge, the current study demonstrated for the first time the overall prevalence of carbapenemase-related genes in recent extraintestinal *E*. *coli* isolates in Abadan, southwest Iran. The current study investigated 152 clinical *E*. *coli* isolates. The majority of strains were retrieved from urine (84.9%) and blood (5.9%). Similarly, in the previous study performed by Tian et al. in China, the *E*. *coli* strains were mainly isolated from urine and blood samples [15]. This research demonstrated a total carbapenem resistance of 18.4% among clinical isolates of *E*. *coli*, which was higher than the rate reported from a study conducted by Zowawi et al. in the gulf cooperation council countries [30]. In our study, the non-susceptibility rates against four tested carbapenems including doripenem, meropenem, imipenem, and ertapenem varied from 13.1% to 23.7%. Antibiotic susceptibility testing showed that the less effective carbapenem in the present study was ertapenem, while most of our isolates were sensitive to doripenem. In contrast to these findings, Manohar et al. from India reported a lower resistance rate for ertapenem compared to meropenem and imipenem in clinical *E*. *coli* isolates [31]. In another study by Sharahi et al. from Iran who investigated 113 clinical *E*. *coli* isolates, 43.4%, 49.6%, 61.9%, and 73.5% of them were resistant against ertapenem, doripenem, meropenem, and imipenem respectively [32]. One other noteworthy finding of the present study was the high frequency of MDR *E*. *coli* (68.4%) which was higher than the previously shown statistics in India (29.6%) [33], Iran (23.9%) [32], and Egypt (58.3%) [11]. These discrepancies in the findings may be due to various reasons, including differences in the geographical area of the research, variation in the pattern of antibiotic prescribing, and the lack of a comprehensive monitoring program for the appropriate usage of antibiotics in some countries.

In this study, according to the results of phenotypic MHT, only 12 (7.9%) isolates showed positive tests and were confirmed as carbapenemase producers, which was in agreement with the report of Khan et al. [34]. Nonetheless, a lower prevalence of carbapenemase-producing *E*. *coli* strains was reported in Egypt [35], as well as in China [15]. MHT results of the current study were further confirmed by PCR assay, and it was found that *bla*_OXA-48_, *bla*_IMP_, and *bla*_NDM_ were existence in 58.3%, 41.7%, and 8.3% of isolates, respectively. Previous studies confirmed the high prevalence of *bla*_OXA-48_. Al-Agamy et al. [36] reported a similar frequency of *bla*_OXA-48_ (60%), while Solgi et al. [37] recorded a higher prevalence rate of *bla*_OXA-48_ (82.8%) and *bla*_NDM_ (31%) than our study. The spread of *bla*_OXA-48_-containing strains has recently been reported in parts of Western Europe and North Africa [38], although it is notable that Turkey is thought to be the major reservoir [39]. On the other hand, various reports from the Middle East [40], Balkans [41], and the Indian subcontinent [42] have demonstrated that these areas could be considered the primary reservoirs for *bla*_NDM_ producers. Therefore, considering that Iran is in the corridor of population transport between Pakistan, Iraq, and Turkey, it can be concluded that at least some carbapenemase-producing strains in Iran, might be originated from these countries, the proof of which requires more comprehensive research. Interestingly, one of the strains isolated from a patient’s bedsore sample not only had a MIC>128 μg/ml and was resistant to all four carbapenem antibiotics tested, but also contained *bla*_OXA-48_ and *bla*_IMP_ simultaneously. However, the *bla*_VIM_ and *bla*_KPC_ genes were not detected in any carbapenem non-susceptible isolates. Several mechanisms for the emergence of MDR strains have been described, some of the most important of which are: 1) association among resistance genes; Antibiotic resistance genes could well be genetically linked if they occur on the same chromosomal region or mobile element, and hence tend to be transported together. 2) Horizontal gene transfer; this mechanism usually occurs for antimicrobials in the same class due to mutations in penicillin-binding proteins as well as beta-lactamases. In addition, it may also occur for various antibiotics in different classes, because the efflux pumps impact a variety of antibiotics in different species. 3) Antibiotic exposure; It occurs mainly due to the routine and inappropriate use of combination therapy by patients and repeated treatment failure [11, 43].

In the current study, phylotype B2 (41.7%) was detected as the predominant group among the carbapenemase-producers. In accordance with our results, Ortega et al. were reported the majority (26.5%) of carbapenemase-producing clinical isolates belonged to phylogroup B2 [17]. Nonetheless, unlike our findings, El-Shaer et al. found that carbapenemase positive clinical isolates were mainly classified as phylogroup C (50%) [44]. This study showed that *E*. *coli* isolate, harbored a wide range of virulence factors genes for ExPEC, encoding siderophores (*iutA*, *aer*, *iroN*), adhesins (*fimA*, *afa*, *papGIII*, *papC*, *sfa*), and toxins (*cnf1*, *hly*, *sat*). In this study, the overall frequency of virulence genes ranged from 6.6% for *hly* to 75% for *fimA*. Type 1 fimbriae, encoded by the *fimA* gene, is associated with biofilm formation and commonly found in most of the *E*.*coli* isolates, conferring as an important virulence factor [18]. Similar to our study, Nojoomi et al. [45] reported that *fimA* was the most frequent virulence gene in clinical *E*.*coli* isolates. Besides, Johnson et al. reported that phylogroups B2 and D were predominant groups among ExPEC clinical isolates, and the frequency of virulence-associated genes varied from 0.4% to 98% [46]. As shown in Table 6, out of a total of 12 carbapenem-resistant strains, 11 isolates (91.7%) and 8 (66.7%) were positive for *fimA* and *iutA*, respectively. The majority (10/11) of the investigated virulence-associated genes, occurred more frequently among carbapenemase producers than non-producers, with statistically significant differences for three genes: *fimA*, *iutA*, and *cnf-1*. It was remarkable that carbapenem-resistant strains isolated from wound specimens had more virulence factor genes than other clinical strains and belonged to phylogroup B1. The lack of molecular typing and sequencing of virulence genes and carbapenems resistance genes were the study’s some limitations.

## Conclusions

Antimicrobial resistance, and in particular resistance to carbapenems, which are often prescribed as a last resort to treat infections, is spreading alarmingly. This work was the first report in Abadan which adds to our knowledge of the frequency of carbapenemase-producing *E*. *coli* isolates, as well as their virulome profiles. According to our findings, doripenem was the most useful carbapenem for the treatment of *E*. *coli* infections. Furthermore, the significant and worrying frequency of MDR-*E*. *coli* underscores the necessity for a surveillance program to restrict the spread of these strains in our region. Among carbapenemase genes, *bla*_OXA-48_, *bla*_IMP_, and *bla*_NDM_ were existences, which were associated with high MIC levels (4 to 128 μg /ml). Virulome analysis of the isolates revealed that the genes involved in adhesin and iron acquisition, especially *fimA* and *iutA* were prevalent in the carbapenems-resistant strains. The emergence of carbapenemase-producing strains encoding various virulence factors is a concern for the treatment of the infections, and proper implementation of monitoring programs is crucial for limiting their dissemination.

## Supporting information

S1 Data (XLSX)

S2 Data (XLSX)

S3 Data (XLSX)

## Abbreviations

CLSI: Clinical and Laboratory Standards Institute
ExPEC: Extraintestinal pathogenic *E*. *coli*
MDR: Multidrug-resistant
MHT: Modified Hodge test
MIC: Minimum inhibitory concentration
